# IFN-γ and CD38 in Hyperprogressive Cancer Development

**DOI:** 10.3390/cancers13020309

**Published:** 2021-01-15

**Authors:** Stefania Angelicola, Francesca Ruzzi, Lorena Landuzzi, Laura Scalambra, Francesco Gelsomino, Andrea Ardizzoni, Patrizia Nanni, Pier-Luigi Lollini, Arianna Palladini

**Affiliations:** 1 Laboratory of Immunology and Biology of Metastasis, Department of Experimental, Diagnostic and Specialty Medicine (DIMES), University of Bologna, 40126 Bologna, Italy; stefania.angelicola2@unibo.it (S.A.); francesca.ruzzi2@unibo.it (F.R.); laura.scalambra2@unibo.it (L.S.); arianna.palladini@unibo.it (A.P.); 2 Laboratory of Experimental Oncology, IRCCS Istituto Ortopedico Rizzoli, 40136 Bologna, Italy; lorena.landuzzi@ior.it; 3 Divisione di Oncologia Medica, IRCCS Azienda Ospedaliero-Universitaria di Bologna, 40138 Bologna, Italy; francesco_gelsomino@aosp.bo.it (F.G.); andrea.ardizzoni@aosp.bo.it (A.A.)

**Keywords:** hyperprogression, hyperprogressive disease, cancer, immune checkpoint inhibitors, immunotherapy, IFN-γ, CD38, macrophage, tumor microenvironment

## Abstract

**Simple Summary:**

Hyperprogressive disease (HPD) is a pattern of paradoxical tumor progression that has been reported in patients treated with immune checkpoint inhibitors (ICIs). Although a large number of studies have investigated HPD and several associated factors have been reported, the mechanisms that drive the acceleration of tumor growth remain unknown. In this review, we discuss the possible role of IFN-γ and CD38 in the development of HPD, and we report the main findings in the scientific literature on the signaling pathways in which these factors take part, and their involvement in response and resistance to ICI therapy.

**Abstract:**

Immune checkpoint inhibitors (ICIs) improve the survival of patients with multiple types of cancer. However, low response rates and atypical responses limit their success in clinical applications. The paradoxical acceleration of tumor growth after treatment, defined as hyperprogressive disease (HPD), is the most difficult problem facing clinicians and patients alike. The mechanisms that underlie hyperprogression (HP) are still unclear and controversial, although different factors are associated with the phenomenon. In this review, we propose two factors that have not yet been demonstrated to be directly associated with HP, but upon which it is important to focus attention. IFN-γ is a key cytokine in antitumor response and its levels increase during ICI therapy, whereas CD38 is an alternative immune checkpoint that is involved in immunosuppressive responses. As both factors are associated with resistance to ICI therapy, we have discussed their possible involvement in HPD with the conclusion that IFN-γ may contribute to HP onset through the activation of the inflammasome pathway, immunosuppressive enzyme IDO1 and activation-induced cell death (AICD) in effector T cells, while the role of CD38 in HP may be associated with the activation of adenosine receptors, hypoxia pathways and AICD-dependent T-cell depletion.

## 1. Introduction

Cancer immunotherapy aims to strengthen the immune system against tumors. The introduction of immunotherapy into clinical practice has provided clinicians with an innovative tool for the treatment of various solid and hematologic malignancies [1]. One of the most successful strategies is the administration of immune checkpoint inhibitors (ICIs), which are a wide range of monoclonal antibodies directed toward immune checkpoint (IC) proteins that are expressed on tumor cell and/or immune cell surfaces. The targeting of ICs reverses tumor-mediated immunosuppression and awakens immune responses [2]. The use of ICIs, either as monotherapy or combo-therapy, has shown favorable outcomes and remarkably long-term responses in patients with a large variety of cancer types, especially malignant melanoma and lung cancer [3,4,5]. The Food and Drug Administration (FDA) has so far approved seven ICIs that target cytotoxic T-lymphocyte antigen 4 (CTLA-4), programmed death-1 (PD-1) and PD-ligand 1 (PD-L1) for the treatment of several tumor types [5]. Nevertheless, response rates, according to the Response Evaluation Criteria in Solid Tumors (RECIST) version 1.1, for IC blockade in patients with solid tumors, range from 18 to 40% [6,7], as the beneficial clinical effects of ICI therapy are not long-lasting in some cases [8]. Moreover, the unique mechanism of action of ICIs leads to unconventional responses, making IC blockade harmful to a subset of patients. Among these novel responses, the most relevant, in terms of negative clinical outcome, is hyperprogressive disease (HPD), a paradoxical acceleration of tumor growth induced by ICI therapy [8]. 

### 1.1. Clinical Evidence of Hyperprogression and Associated Factors

HPD was first reported in retrospective studies in which patients showed rapid disease progression after the initiation of ICI therapy. Evidence suggests that a subset (4–29%) of cancer patients develops HPD [9], with a particular reference to patients with non-small-cell lung cancer (NSCLC) (from 8 to 21%), advanced gastric cancer (AGC) (from 10 to 29%), head and neck squamous cell carcinoma (HNSCC) (29%) and melanoma (9%) [9,10,11,12,13,14,15]. After Champiat et al. had introduced the concept of hyperprogression (HP) in a retrospective study of cancer patients receiving PD-1/PD-L1 inhibitors [12], several groups assessed the phenomenon and proposed different criteria for its definition, resulting in notable variations in HPD rates (Table 1). The lack of a univocal definition makes the phenomenon controversial and adds to the difficulty of comparing different studies [16]. Nevertheless, Kas et al. have recently suggested an optimized and homogenized definition of HPD, based on the previously used criteria [17]. 

Older age (≥65) was one of the factors identified by various studies in association with an increased risk of developing HPD during ICI therapy [12,22,23,24]. Age-related immune dysfunction (ARID) leads to a decrease in the quantity and activity of T cells [25,26], which are essential for the success of ICI therapy. Previous irradiation was also associated with higher incidence of hyperprogression [14]. Alterations in the tumor infiltrate after radiotherapy, which promotes the production of neoantigens, may facilitate rapid progression in the irradiated area [27]. In a retrospective study involving patients with NSCLC treated with ICIs, hyperprogression was significantly associated with the presence of more than two metastatic sites before the treatment [13]. Mouse double minute homolog (MDM2/MDM4) amplification and epidermal growth factor receptor (EGFR) alterations were indicated as genomic markers of increased risk of hyperprogression [19,28]. Furthermore, patients with oncogene-addicted NSCLC, e.g., ALK, EGFR and STK11, did not benefit from IC blockade therapy, probably because of the “cold” nature of these tumors [29,30,31,32,33,34]. Hyperprogression was also associated with elevated serum lactate dehydrogenase (LDH) concentration [10,12,15,35]. High levels of LDH may be indicative of a hypoxic and acid microenvironment, which reduces the functionality of infiltrating T lymphocytes (TILs) and natural killer (NK) cells [36,37].

In contrast, no association between HPD and tumor histology, baseline tumor size and previous lines of therapy has been reported [12,14,19]. Concerning the association of PD-L1 expression with HPD, studies have shown discordant results [38,39,40]. Nevertheless, a significant inverse correlation between PD-L1 expression in tumor cells and HPD has been detected in NSCLC patients [21].

### 1.2. Hypothesized Mechanisms of HPD

**Figure 1:**
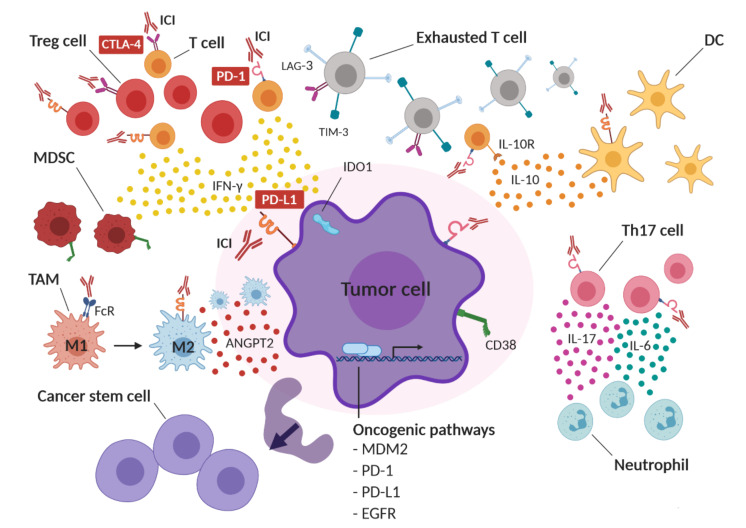
Possible mechanisms of hyperprogressive disease (HPD) in cancer after immune checkpoint blockade therapy. ICI therapy may functionally activate infiltrating regulatory T cells (Tregs), leading to an immunosuppressive tumor microenvironment. At the same time, compensatory upregulation of alternative immune checkpoints, such as lymphocyte-activation gene 3 (LAG-3), T-cell immunoglobulin and mucin domain-3 (TIM-3) and CTLA-4, on effector T cells after ICI therapy may induce T-cell exhaustion. IC inhibition might also induce the expression of cluster of differentiation 38 (CD38) on tumor cells and IFN-γ-dependent recruitment of CD38-expressing myeloid-derived suppressor cells (MDSCs), resulting in immune suppression. Moreover, ICI therapy may induce the upregulation of the immunosuppressive enzyme indoleamine 2,3-dioxygenase (IDO1) and the increased secretion of immunosuppressive cytokines and soluble molecules, such as interleukin 10 (IL-10), angiopoietin-2 (ANGPT2) and interferon-gamma (IFN-γ) into the tumor microenvironment. IC blockade might also functionally boost T helper 1 (not shown) and T helper 17 (Th17) lymphocytes, resulting in neutrophil recruitment and in an inflammatory immunosuppressive tumor microenvironment enriched in interleukin 6 (IL-6) and interleukin 17 (IL-17). In addition, ICIs may bind to Fc receptors (FcR) on tumor-associated macrophages (TAMs), resulting in a shift from M1 to an immunosuppressive M2 phenotype. IC blockade may also induce an increase in cancer stem cells (CSC) and may activate oncogenic pathways, such as MDM2, PD-1, PD-L1 and EGFR, thus promoting tumor proliferation. DC: dendritic cell.

The mechanisms underlying hyperprogression after ICI therapy are still unknown; however, alterations in T-cell subpopulations, cytokine secretion, inflammation and tumor cells have been proposed (Figure 1).

ICs, such as CTLA-4, PD-1 and PD-L1, can be overexpressed on immunosuppressive tumor-specific regulatory T (Treg) cells [41,42,43]. A rapid expansion of forkhead box P3^+^ (Foxp3^+^) Treg cells has been observed in the tumors of HPD patients with advanced gastric cancer treated with nivolumab. Moreover, PD-1 blockade enhances the proliferation and suppressive activity of human Treg cells in vitro [11]. 

IC blockade therapy can also induce compensatory mechanisms, leading to T-cell exhaustion, local immune suppression and tumor escape. Two independent studies have reported the compensatory upregulation of ICs, including lymphocyte activation gene-3 (LAG-3), T-cell immunoglobulin and mucin domain-3 (TIM-3) and CTLA-4, on CD8^+^ T cells after PD-1 blockade in immunocompetent murine models of ovarian cancer and lung adenocarcinoma [44,45]. ICI therapy can also upregulate cluster of differentiation 38 (CD38) on tumor cells, leading to immune suppression and resistance to therapy [46]. In addition, the aberrant expansion of peripheral exhausted CD4^+^ memory T cells has been reported to occur after the first administration of anti-PD-1/PD-L1 antibodies in patients with HPD, unlike non-HPD patients [47].

The compensatory immune response triggered by IC blockade can induce the production of immunosuppressive cytokines and other soluble mediators. In preclinical studies, PD-1/PD-L1 blockade increased IL-10 secretion by tumor infiltrating dendritic cells (DCs) and upregulated PD-L1 on DCs, leading to tumor immune escape [48]. In addition, tumor-specific CD8^+^PD-1^+^ T cells, in patients with advanced melanoma under PD-1 blockade therapy, overexpressed the IL-10 receptor (IL-10R). The inhibition of IL-10 strengthened the effect of anti-PD-1 antibodies in expanding tumor-specific CD8^+^ T cells, and thus reinforced their antitumor action [49].

Angiopoietin-2 (ANGPT2) has been proposed as a predictive and prognostic marker in ICI-treated patients with advanced melanoma. High levels of ANGPT2 in serum before treatment were associated with reduced response and/or overall survival and with higher levels of immunosuppressive M2 macrophages in ICI-treated patients [50,51]. 

Aberrant inflammation that is caused by increased T helper 1 (Th1) and Th17-dependent secretion of inflammatory cytokines, such as IFN-γ, IL-6 and IL-17, which are associated with neutrophil recruitment, has been observed in patients with prostate cancer and melanoma that were treated with PD-1/PD-L1 inhibitors [52]. Interestingly, neutrophil depletion and IL-6 blockade resulted in more effective antitumor immune responses in mouse models [53,54,55]. Moreover, the interaction between the Fc domain of ICIs and Fc-receptors (FcR) induces macrophage reprogramming, from the M1 to M2 phenotype, in patients with HPD [21].

Tumor-specific non-lytic CD8^+^ T cells induce the overexpression of PD-L1 and IDO1, which are associated with adaptive immune resistance and stemness phenotype in tumor cells [56]. 

MDM2 is an oncoprotein that is involved in the degradation and inhibition of p53. The amplification of this gene has frequently been observed in HPD patients [19]. IFN-γ-induced interferon regulatory factor 8 (IRF-8) induces MDM2 overexpression by binding to its promoter [19,57].

The activation of the PD-1 axis on T cells reduces T-cell proliferation [58], while PD-1 inhibition on neoplastic T cells accelerates tumor growth [59,60,61]. PD-1 expression on tumor cells drives melanoma tumorigenesis via PD-1/PD-L1 interaction [62]. Conversely, preclinical data suggest that PD-1 blockade may increase tumor growth by interfering with the PD-1-dependent upregulation of proapoptotic proteins, e.g., BIM, p15INK4 and cyclin-dependent kinase 2 [63].

PD-1/PD-L1 interaction transmits antiapoptotic signals to cancer cells, leading to resistance to T-cell-mediated cytolysis and Fas-mediated apoptosis. The elimination of the intracellular domain of PD-L1 ablated cancer resistance to immune response, leading to tumor regression [64]. In addition, PD-L1 can transmit protumorigenic intracellular signaling, even in a PD-1-independent manner, by interfering with the autophagy process or activating mTOR [65,66]. PD-L1 expression also protects tumor cells from IFN-antitumor action by inhibiting STAT3-caspase 7 signaling [67]. Finally, activating mutations of EGFR drive PD-L1 expression on several tumor types, including NSCLC, HNSCC and breast cancer [68]. The EGFR-dependent mechanism of PD-L1 regulation involves post-translational modifications. Indeed, the inhibition of EGFR signaling destabilizes PD-L1 expression in mouse models, thus enhancing the therapeutic efficacy of PD-1 blockade [69,70]. 

## 2. Role of IFN-γ in HPD

IFN-γ is a major regulatory and effector cytokine predominantly produced by T and NK cells in response to inflammatory and immune stimuli. In the tumor microenvironment (TME), IFN-γ, mainly produced by TILs, is a key player in tumor immunosurveillance. The antitumor action includes antiproliferative, antiangiogenic and proapoptotic effects [71,72,73,74,75], in addition to the upregulation of major histocompatibility complex (MHC) class I molecules on tumor cells [76,77]. Moreover, IFN-γ activates CD8^+^ cytotoxic T lymphocytes, CD4^+^ Th1 cells, NK cells, DCs and macrophages, and stimulates the latter to switch towards the tumoricidal and proinflammatory M1 phenotype [77,78,79]. Conversely, IFN-γ inhibits Treg cell differentiation and function [80]. 

On the other hand, IFN-γ exerts a paradoxical immunosuppressive role that supports tumor progression and dissemination [81,82,83]. The activation of the IFN-γ receptor (IFNGR) on tumor cells activates the JAK/STAT signaling pathway, resulting in PD-L1 upregulation [84,85,86]. Nevertheless, alterations in the JAK/STAT pathway have been frequently associated with resistance to ICI therapy [87,88,89]. For instance, human melanoma cell lines with loss-of-function mutations in either JAK1 or JAK2 do not express IFN-γ-response genes after IFN-γ exposure. The analysis of the transcriptome of advanced melanoma under ICI therapy has highlighted an association between clinical response to treatment and the expression of IFN-γ-response genes involved in MHC class I and II upregulation [90]. Resistance to ICI therapy may therefore be due both to the incapacity of tumor cells to induce the full set of IFN-γ-response genes and to the loss of sensitivity to IFN-γ signaling. Interestingly, prolonged IFN-γ receptor signaling in tumor cells can also mediate resistance to ICIs through epigenomic changes in the JAK/STAT pathway [91].

IFN-γ can even support tumorigenesis by influencing the TME. The IFN-γ signaling pathway can indeed increase angiogenesis in the TME by inhibiting the expression of vascular endothelial growth inhibitor (VEGI) [92]. IFN-γ also suppresses the action of immune effector cells via the upregulation of immunosuppressive cytokines, including IL-21, IL-27 and IL-35 [93,94,95,96,97,98], and by the recruitment and differentiation of Treg cells and MDSCs [99,100,101,102,103,104,105,106,107].

All of the above-reported examples highlight the role that IFN-γ plays in tumor resistance to ICI therapy. Based on this evidence, it is reasonable to assume that IFN-γ is worthy of investigation in the context of HPD. 

### 2.1. IFN-γ and Inflammasome

Several studies reported a negative correlation between MDSCs and the response to ICIs [108,109,110], leading to the suggestion that MDCSs can be a negative predictive marker for ICI therapy [111]. A few case reports noted a correlation between HPD development and the number of MDSCs in the peripheral blood and in the TME [112,113]. Moreover, the recruitment of granulocytic MDSCs to the TME following ICI therapy, through the IFN-γ-dependent activation of the inflammasome pathway in cancer cells, has been reported [114]. PD-L1 upregulation by IFN-γ after ICIs and the activation of the PD-L1 intrinsic signaling pathway in tumor cells trigger the activation of NLR family pyrin domain containing 3 (NLRP3), which leads to the downstream activation of the heat-shock proteins 70 (HSP70)/Toll-like receptor 4 (TLR4) signaling pathway and Wnt5a production. This signaling cascade ultimately leads to C-X-C motif chemokine ligand 5 (CXCL5) release, resulting in chemokine-dependent recruitment of polymorphonuclear-like MDSCs [114]. PD-L1 triggers NLRP3 activation by repressing STAT3, which is a transcription factor involved in IFN-cytotoxicity. Mutations in the intracytoplasmic DTSSK domain of PD-L1, which is a conserved sequence that acts as a negative regulator of PD-L1 functions, lead to hyperactive PD-L1 molecules in human tumors, enhancing the capacity of the PD-L1 intracellular pathway to interfere with STAT3 expression and phosphorylation [67]. Thus, in patients carrying mutations in the intracytoplasmic domain of PD-L1, the molecules of the inflammasome may be further augmented by PD-L1 upregulation after IFN-γ secretion in response to ICIs, eventually leading to HPD. Interestingly, a mutational analysis performed on tumors after pembrolizumab treatment highlighted the presence of missense or indel mutations in genes involved in the negative regulation of NLRP3 activation and inflammasome pathway [113], including the caspase recruitment domain (CARD8 and CARD11), protein flightless-1 homolog (FLII) and nuclear factor erythroid 2-related factor 2 (NFE2L2) [115,116,117,118]. Moreover, hyperprogressive tumors show mutations in NOTCH1, which seems to be involved in NLRP3 activation and inflammasome pathway [19,113,119,120,121]. In addition, the mechanism of MDSC recruitment in HPD may also be related to the impairment of effector T-cell activity, resulting in the expansion of Treg cells, the inhibition of NK cells and the secretion of immunosuppressive cytokines [122]. IFN-γ-mediated recruitment of MDSCs in the TME may therefore be a relevant aspect of HPD.

### 2.2. IFN-γ and IDO1

Paracrine Wnt5a signaling is also involved in DC upregulation and enzymatic activity of IDO1, resulting in the promotion of DC-mediated Treg differentiation [123,124]. IDO1 is a cytosolic enzyme that contributes to immune regulation by inducing metabolic changes in the local microenvironment. The enzyme catalyzes the rate-limiting step of tryptophan metabolism, which converts tryptophan (trp) into the downstream catabolite kynurenine (kyn). IDO1 is physiologically expressed by professional antigen-presenting cells (APCs), as well as by epithelial cells, the vascular endothelium and peripheral lymphoid organs, and acts as a peripheral IC, contributing to host defense against infection, peripheral immune tolerance, inhibition of local inflammation and autoimmunity [125,126]. Tumor cells use IDO1 expression as a mechanism of immune escape [127,128,129]. Trp depletion indeed results in a blockade of T-cell protein synthesis [130,131], while kyn and its derivatives induce PD-1 expression on activated T cells, and the differentiation of Treg cells and tolerogenic DCs through aryl hydrocarbon receptor (AhR) activation [132,133,134,135]. In addition, IDO1 expression is induced, as is that of PD-L1, when some degree of inflammation occurs in the tumor, e.g., the presence of proinflammatory mediators, such as IFN-γ, as a mechanism of adaptive resistance against infiltrating T cells [136,137,138]. This aspect clearly has negative implications for ICI therapy, since increased IDO1 activity may increase tumor-infiltrating Treg cells, decrease TILs and accelerate tumor growth [139,140,141,142,143]. In NSCLC patients treated with nivolumab, serum kyn/trp ratio was higher in early progressors with intrinsic resistance to anti-PD-1 therapy. Moreover, patients with high kyn/trp ratios showed a progression-free survival of three months, which is very similar to that of hyperprogressive patients in a study by Champiat et al. [12,144]. This evidence suggests that IDO1 induction by IFN-γ after ICI therapy may counteract the effectiveness of an otherwise beneficial treatment. Combination treatment with ICIs and IDO1 inhibitors in preclinical studies has been observed to enhance the infiltration and proliferation of effector T cells in the TME [145,146]. However, despite encouraging clinical results in early phase trials [147,148,149], in a randomized phase III study, patients with metastatic melanoma treated with both the IDO1 inhibitor epacadostat and pembrolizumab, had no benefit from the combined therapy, in comparison to pembrolizumab monotherapy [150]. This negative result may, however, be caused by several factors, including limited preclinical data and either the incomplete inhibition of IDO1 or the compensatory expression of other trp-degrading enzymes [151]. Further clinical studies are therefore needed to understand whether this combined therapy may have therapeutic potential. 

Interestingly, IDO1 upregulation is inversely correlated with p53 [152], whose expression can be suppressed by IDO1 via the c-Jun N-terminal kinase (JNK) pathway [153]. The downregulation of p53 has already been suggested as a HPD mechanism in patients with MDM2 amplification [19]. Moreover, IDO1 expression is modulated by transforming growth factor-beta (TGF-β) via the Fyn-dependent phosphorylation of immunoreceptor tyrosine-based inhibition motifs (ITIMs) in IDO1, and the activation of the NF-κB pathway, leading to a tolerogenic phenotype in DCs [154]. TGF-β can also activate the JNK pathway through TGF-β activated kinase 1 (TAK1) and c-Jun phosphorylation [155,156]. The TGF-β signaling pathway has been found to be transcriptionally upregulated in HPD tumors following IC blockade, as compared to treatment-naive tumors [113]. Therefore, HPD patients with IDO1-expressing tumors may present a hyperactivated JNK pathway which may result in p53 suppression. Moreover, post-therapy HPD tumors have also displayed transcriptional upregulation of PI3K/AKT and MAPK/ERK pathways, and it has been shown that PI3K and MAPK oncogenic mutations can favor constitutive IDO1 expression on tumor cells [113,157,158]. 

### 2.3. IFN-γ and Activation-Induced Cell Death

A further hypothesis that involves IFN-γ focuses on the differential immunological actions of IC blockade that occur depending on tumor burden. The combination of anti-CTLA-4 and anti-PD-1 therapy in mice with high tumor burden (HTB) leads to improved tumor control and to the generation of more activated antigen-specific T cells, as compared to mice with low tumor burden (LTB), in which combination treatment compromised antitumor immune response, inducing the loss of antigen-specific T cells [159]. This finding was supported by retrospective clinical data from metastatic melanoma patients who received either monotherapy or combination therapy. Patients treated with dual IC blockade showed significantly lower response rates than those treated with monotherapy in low disease settings, but not in higher disease settings. The detrimental effect of combined therapy in the LTB state was associated with higher IFN-γ production, which was responsible for tumor-reactive CD8^+^ T-cell apoptosis via activation-induced cell death (AICD). AICD physiologically takes place in the early CD4^+^ and CD8^+^ T-cell priming stage, and leads to cell apoptosis to prevent immune hyperactivation. IFN-γ signaling is the key factor in activating this process, together with IL-2 [160]. The induction of IFN-γ secretion after dual-blockade treatments can promote the apoptosis of tumor-reactive CD8^+^ T cells in the LTB setting, limiting the formation of effector memory antitumor responses. In the HTB state, prolonged antigen exposure may lead to T cells with a markedly exhausted phenotype, which may be more prone to reinvigoration after IC blockade. By contrast, in LTB or in the early tumor setting, short-term antigen exposure may be unable to induce a fully exhausted phenotype in T cells. Therefore, the activation of T-cell-receptor signaling against tumor antigens, in combination with dual IC blockade, may result in immune hyperactivation, triggering the AICD process. These findings appear to indicate that the paradoxical effect of IFN-γ in tumor response might derive from the differential exhaustion status of T cells in response to ICIs. Although the mechanisms underlying AICD have not yet been fully understood, Fas seems to be the major death receptor responsible for triggering the AICD pathway in CD4^+^ T cells [161]. Moreover, STAT1 and caspase 8, which are activated by the IFN-γ pathway, may be involved in the process [160]. The possible role of the activation of the AICD pathway by IFN-γ in HPD is supported by a study in which two hyperprogressive patients displayed depletion of the immune-cell populations involved in tumor clearance, including monocytes, central memory CD4^+^ T cells, NK cells and activated DCs [113]. In these patients, anti-PD-1 therapy had probably induced an accelerated AICD process in the antitumor activating lymphocytes, as suggested by the activation of apoptosis gene sets and the upregulation of marker genes in the bcl-2 pathway after treatment. These studies suggest that, in some patients, ICI therapy may be responsible for excessive activation of the immune response, which could trigger regulatory mechanisms and hinder therapeutic antitumor effects. 

**Figure 2:**
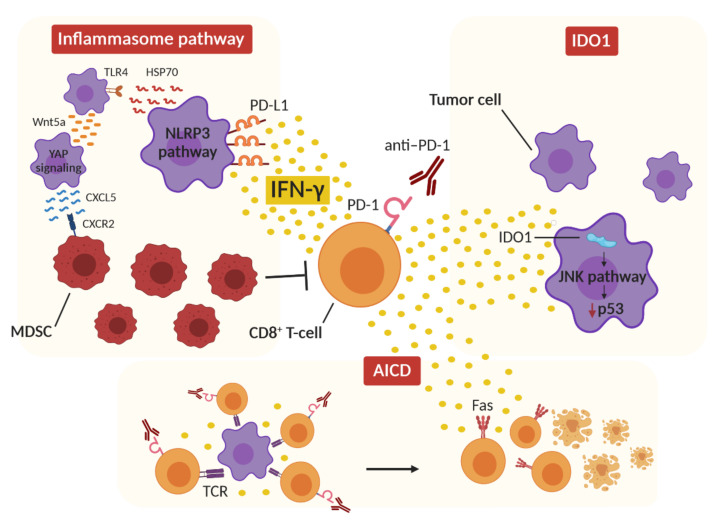
Proposed IFN-γ-dependent mechanisms of hyperprogression. The release of IFN-γ from CD8^+^ T cells after ICI therapy can activate the inflammasome pathway by upregulating PD-L1 expression on tumor cells and activating NLRP3 signaling, resulting in immunosuppressive MDSC recruitment in the tumor microenvironment. At the same time, IFN-γ can induce IDO1 activity in tumor cells, which activates the JNK pathway, leading to p53 downregulation and tumor growth. Finally, the concomitant stimulation of tumor-specific CD8^+^ T cells by ICI therapy and T-cell-receptor (TCR) activation results in a hyperactivated immune environment in which IFN-γ triggers the activation-induced cell death (AICD) mechanism and T-cell Fas-mediated apoptosis.

In conclusion it may be suggested that IFN-γ contributes to HPD onset in predisposed patients via the induction of the inflammasome pathway and consequent MDSC recruitment, the induction of IDO1 activity, which may result in the downregulation of p53 in tumor cells, and the activation of AICD, which leads to T-cell depletion (Figure 2).

## 3. Role of CD38 in HPD

CD38 is a multifunctional ectoenzyme that is widely expressed on the surface of multiple immune populations, including T cells, NK cells, DCs and myeloid-derived cells. This molecule, which performs both enzyme and receptor activity, is involved in the regulation of intracellular Ca^2+^, cell adhesion, infection, tumorigenesis and immunosuppression [162,163]. As far as the enzymatic activity of CD38 is concerned, it acts as a NADase, catalyzing nicotinamide adenine dinucleotide (NAD^+^) hydrolysis and creating immunosuppressive byproducts such as adenosine diphosphate-ribose (ADPR) and cyclic ADPR (cADPR). Moreover, under acidic conditions, CD38 catalyzes the formation of nicotinic acid adenine dinucleotide phosphate (NAADP) from nicotinamide adenine dinucleotide phosphate (NADP^+^) [163]. CD38 NADase activity contributes to T-cell exhaustion via the reduction of NAD^+^ levels in T cells. Exhausted T cells indeed show high PD-1 and CD38 expression [164,165].

Concerning the function of CD38 as a receptor, its short cytoplasmatic tail makes CD38 unable to activate a downstream signaling pathway [166]. CD38 indeed depends on other signaling receptors and cell-surface molecules, such as CD31, which is an adhesion protein that is mainly expressed on endothelial cells. The interaction between CD38 and CD31 induces dynamic interactions between CD38^+^ immune cells and endothelial cells, promoting cell migration and homing [167,168,169]. In T cells, however, the interaction between CD38 and CD31 induces cell activation, which is mediated by the CD38-dependent phosphorylation of the intracellular proteins involved in T-cell-receptor (TCR)/CD3 signaling pathway and MAPK-pathway activation [170]. CD38 ligation in peripheral blood mononuclear cells (PBMCs) induces the expression of IL-1β, IL-6, IL-10 and IFN-γ [171], while in NK cells, CD38 association with CD16 promotes degranulation and IFN-γ production [172,173]. In B cells, CD38 induces either proliferation or apoptosis, depending on differentiation stage, thanks to its association with B-cell-receptor (BCR) downstream pathway [174].

Despite the cooperation of CD38 in effector cytotoxic responses, the enzyme is involved in immune escape and tumor growth. CD38 has been widely investigated in chronic lymphocytic leukemia (CLL) and multiple myeloma (MM). In CLL, CD38 is a marker of both poor prognosis and aggressive tumor phenotype [175,176]. In CLL cells, CD38 expression, which works in synergy with the BCR/CD19 complex, is associated with greater proliferative and antiapoptotic activity as well as with enhanced migratory ability [177,178,179]. CD38 is highly expressed on MM cells as well [180,181]. This finding has made CD38 a target for therapeutic antibodies in MM, including daratumumab, a human anti-CD38 antibody that has been approved by the FDA both as monotherapy and in combination with standards of care, and isatuximab, a human anti-CD38 antibody that has recently been approved in combination with pomalidomide and dexamethasone [182,183]. Both daratumumab and isatuximab have been shown to effectively induce myeloma cell death via Fc-dependent mechanisms of action and the depletion of CD38^+^ immunosuppressive cells [183,184,185,186,187,188]. 

In solid tumors, CD38 is involved in immune evasion and tumor progression. In the TME, CD38 can be upregulated on MDSCs, inducing a more immature and immunosuppressive phenotype [189]. In gliomas, the expression of CD38 in the TME has been associated with F4/80 infiltrating microglia and macrophages, which favor tumor growth, and suggest that CD38 may play a role in the recruitment and survival of these immune subpopulations [190]. Other studies have demonstrated that CD38 has a role in tumor growth that is independent of the expression of CD38 on immune cells, and is rather due to its expression on tumor cells. The knockout of CD38 on tumor cells reduced the invasion and progression of tumors in nude mice models, while the expression of CD38 by tumor cells enhanced in vitro and in vivo growth [191,192]. Furthermore, CD38, when expressed on tumor cells, can inhibit CD8^+^ T cells via adenosine production and the activation of adenosine receptor signaling on T cells [46]. Based on the above-reported evidence, it can be stated that CD38 exerts a protumorigenic role, both through its enzymatic activity and its receptor functions, by activating downstream pathways in tumor cells that enhance their proliferative, antiapoptotic and migratory ability, and promoting immune evasion through the recruitment of immunosuppressive cells and T-cell exhaustion.

CD38 expression has also been correlated with resistance to ICI therapy. PD-1/PD-L1 blockade has been reported to upregulate CD38, not only on CD8^+^ cytotoxic T cells, but also on tumor cells [46,193]. CD38 expression can be induced in monocytes, bone-marrow progenitor cells and CLL cells by IFN-γ [194,195,196]. Treatment with the anti-CD38 antibody in mice that are resistant to IC blockade therapy inhibits tumor growth, enhances effector CD8^+^ and CD4^+^ T-cell responses and reduces CD4^+^ Treg cells and MDSCs [46]. CD38 expression in tumors has been recognized as a biomarker of poor response to ICI therapy [197].

### 3.1. CD38 and Adenosine Receptor Activation

CD38 mediates IC-blockade resistance through the modulation of adenosine receptor signaling in the TME, which leads to the inhibition of T-cell proliferation and function [46]. As briefly mentioned above, CD38 enzymatic activity includes the production of ADPR and cADPR. ADPR can be further metabolized by CD203 and CD73 to produce adenosine, which is implicated in immune suppression through the activation of adenosine receptors (ADORA) [198]. The activation of ADORA on tumor cells regulates cell proliferation, apoptosis, cytoprotection and migration [199,200]. In particular, the adenosine A2A receptor (ADORA2a) has been found to be overexpressed in HNSCC [201,202] and in several melanoma and breast cancer cell lines [203,204,205]. The ADORA2a downstream pathway inhibits JAK/STAT signaling, either by preventing phosphorylation or inducing the proteasomal degradation of STAT proteins [206]. ADORA2a-driven STAT degradation may be involved in HPD along with the JAK/STAT dysfunctions that were previously described in patients who are resistant to IC blockade therapy. Although CD38 upregulation has not been directly linked to hyperprogressive tumors, it is interesting to highlight the reported mutations of NOTCH genes in HPD patients [19,113] and the correlation between NOTCH signaling activation and CD38 expression [207]. Furthermore, ADORA2a physically interacts with the fibroblast growth factor receptor (FGFR) and the concomitant activation of these receptors induces the activation of the MAPK/ERK pathway in neural tissue [208]. The amplification of FGF genes and the upregulation of the TGF-β pathway have been reported in hyperprogressive tumors [28,113]. These data suggest that, in hyperprogressive tumors, these alterations may lead to hyperactivation of the presumed ADORA2a-FGFR-dependent pathway, possibly resulting in accelerated tumor growth. Moreover, the expression of ADORA2a by tumor cells can induce the JNK signaling pathway, leading to p53 downregulation [209,210]. Furthermore, the activation of ADORA2a by extracellular adenosine results in the accumulation of intracellular cAMP, which induces the upregulation of inhibitory receptors, such as PD-1, increased Foxp3 expression in CD4^+^ T cells, the induction of effector T-cell anergy and the upregulation of inhibitory cytokines, including TGF-β [211]. Since Treg cells express high levels of CD39 and CD73, which catabolize adenosine triphosphate (ATP) and adenosine diphosphate (ADP) into adenosine, ADORA2a can lead to an immunosuppressive amplification circuit in which increasing amounts of adenosine are created [178]. It can be hypothesized that, in a context of high CD38 activity and, consequently, high adenosine levels in TME, hyperprogressive tumors may display a hyperactivated ADORA2a pathway, which may confer resistance to IFN-γ cytotoxic action, enhance oncogenic pathways, such as MAPK/ERK and TGF-β, and induce a strong immunosuppressive microenvironment through the shift of CD4^+^ T cells towards a Treg phenotype. Hyperprogressive patients have indeed demonstrated a marked increase in effector Foxp3^high^ Treg cells with proliferative capacity compared to non-hyperprogressive patients, who, on the contrary, showed a significant reduction [212]. Adenosine signaling can even have an impact on DC maturation, inducing the secretion of tolerogenic factors such as vascular endothelial growth factor (VEGF), IL-10, TGF-β, arginase and IDO1 [213]. In addition, adenosine signaling triggers the increased expression of other IC pathways, such as PD-1, CTLA-4 and LAG-3, in both effector and Treg cells [214,215]. Finally, adenosine receptor stimulation promotes M2 polarization in tumor-associated macrophages (TAMs), which are known to be involved in HPD [21,216]. These findings demonstrate that exposure to high concentrations of adenosine, which are induced by CD38 activation, can dampen the antitumor function of immune cells, contributing to a more immunosuppressive microenvironment that may interfere with the therapeutic response to ICIs. 

### 3.2. CD38 and Activation-Induced Cell Death

CD38 expression seems to occur in settings with active intratumoral cytolytic immune-cell infiltrates and immune-inflammatory signatures, e.g., Foxp3, CTLA-4, PD-1, PD-L1, LAG-3, TIM-3, IDO1 and C-C Motif Chemokine Ligand 2 (CCL2). Thus, CD38 upregulation may be a consequence of intratumoral T-cell infiltration and associated changes in the cytokine/metabolite milieu after IC blockade [46]. ICI therapy may exert prolonged pressure that may induce CD38 upregulation as a compensatory mechanism of immune regulation. Over time, the immunosuppressive effect of highly CD38-expressing tumor cells may become dominant over the expression of PD-1 and PD-L1, resulting in resistance to treatment. Interestingly, in a study of microglial activation in the central nervous system, CD38 promoted the AICD process via cADPR production [217]. CD38^−/−^ primary microglia was more resistant to AICD than wild-type microglia. Moreover, CD38 can promote nitric oxide production, which is an essential mediator for AICD. Nevertheless, CD38 does not seem to independently regulate the AICD process, since it is not able to regulate caspase 11 expression, which is an important mediator of AICD. It can therefore be stated that CD38 participates in AICD activation, which, however, is also mediated by other pathways. Interestingly, IC blockade therapy induces not only CD38 upregulation, but also increased expression of IRF-1, which upregulates Fas ligand (FasL) transcription by directly binding to its promoter [46,218]. As Fas/FasL signaling is the main mechanism of AICD, it can be concluded that ICI therapy, and CD38 upregulation after treatment, seem to be associated with the activation of AICD, which may in turn lead to the depletion of activated cytotoxic CD8^+^ T cells in the TME and, consequently, tumor growth.

### 3.3. CD38 and Hypoxia

Increased CD38 expression has also been associated with a markedly hypoxic microenvironment, which stimulates angiogenesis events and immunosuppressive actions. Hypoxia upregulates inhibitory signals for antitumor immune response, including PD-L1, IDO1, IL-6 and IL-10, and promotes the polarization of TAMs towards a M2-like phenotype. Moreover, tumor hypoxia recruits Treg cells in the TME via the upregulation of chemokines, while the increased expression of FasL on the tumor endothelial barrier in hypoxic conditions can selectively eliminate effector CD8^+^ T cells. Increased expression of ANGPT2 mRNA in mononuclear cells of untreated CLL patients has been associated with high CD38 expression [219], which influences the expression of hypoxia-inducible factor 1-alpha (HIF-1α) [220]. The overexpression of HIF-1α has been associated with poor prognosis and an aggressive phenotype in several malignant tumors [221,222,223]. HIF-1α takes part in tumor angiogenesis via the regulation of VEGF expression [224,225]. The ability of CD38 to promote HIF-1α expression may be related to the onset of hyperprogression. Post-therapy tumors of HPD patients show somatic mutations in the von Hippel–Lindau tumor suppressor gene (VHL), which is a master regulator of HIF activity [113,226]. Biallelic VHL inactivation in renal cell carcinoma (RCC) is indeed associated with increased HIF-1α, which is responsible for the highly vascular nature of RCC [113,227]. A nonsense mutation in the BRCA1 associated protein 1 (BAP1) gene has been reported in the post-therapy tumor of a hyperprogressive patient [113], and the loss of BAP1 expression was highly correlated with HIF-1α expression in uveal melanoma. It has been proposed that BAP1 may lead to HIF-1α upregulation via the NF-κB pathway [228]. Furthermore, hyperprogressive patients show the transcriptional upregulation of the oncogenic insulin-like growth factor 1 (IGF-1) signaling pathway and somatic mutation in the IGF-binding protein 2 (IGFBP2) gene [113]. Interestingly, a study has reported that HIF-1α overexpression in tumor cells takes part in an autocrine growth factor loop, which involves the HIF-1α-dependent expression of genes encoding IGF-2, IGFBP-2 and IGFBP-3. Therefore, HIF-1α may be involved in tumor growth, resistance to apoptosis and migration via the activation of IGF pathways [229]. HIF-1α is also a driver of VEGF expression in tumors and may help to establish autocrine and paracrine signaling networks in the TME. For instance, VEGF acts as a chemoattractant for CD4^+^Foxp4^+^ Treg cells into the tumor [230]. Moreover, the autocrine signaling of VEGF in tumor cells, through the activation of the VEGF receptor (VEGFR), contributes to tumorigenesis, tumor dedifferentiation and invasion [231,232]. Hyperprogression has been significantly associated with VEGFR2 rs1870377 A/T and A/A polymorphisms [233], which are able to increase VEGF-A binding and activity, inducing increased microvessel density in tumors [234]. Hyperprogressive tumors may show strong binding between VEGF and VEGFR, either on endothelial cells or on tumor cells, leading to increased angiogenesis and rapid tumor growth. The association between CD38 expression and the induction of a hypoxic environment is further confirmed by the strong correlation between higher serum LDH and CD38 expression in CLL patients, in which CD38 expression was also associated with shorter overall survival compared to patients without CD38 expression [235]. Based on the above-reported evidence, the association between CD38 and hypoxic responses may be an important part of HPD development. 

**Figure 3:**
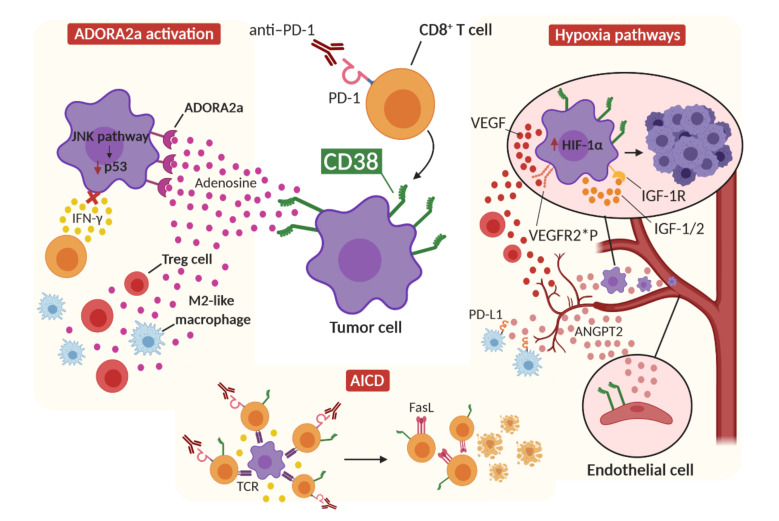
Proposed CD38-dependent mechanisms of hyperprogression. The upregulation and activation of CD38 after IC blockade therapy, together with the action of enzymes that catabolize ATP and ADP (not shown), lead to the release of adenosine into the tumor microenvironment. The activation of ADORA2a receptors on tumor cells by adenosine leads to the blockade of the IFN-γ pathway, making the tumor cell resistant to cytotoxic IFN-γ action, as well as the activation of oncogenic pathways, such as the JNK signaling pathway, which leads to the downregulation of p53. The action of adenosine also increases Treg cells and the polarization of M2 macrophages in the tumor microenvironment. CD38 also promotes the activation of AICD after the hyperactivation of tumor-specific CD8^+^ T cells by ICI therapy, and induces the expression of FasL on T cells. CD38 also induces the release of Angiopoietin-2 from endothelial cells, leading to increased angiogenesis, PD-L1 expression on M2-like macrophages and enhanced tumor invasion. Finally, CD38 induces the expression of HIF-1α in tumor cells, which can, in turn, induce the production of VEGF and IGF factors. VEGF can establish paracrine signaling, promoting the recruitment of Treg cells, and autocrine signaling on tumor cells by binding VEGFR2, promoting tumorigenesis and invasion. The polymorphisms rs1870377 A/T and A/A may make this binding stronger. HIF-1α can also induce the expression of IGF-pathway components, leading to autocrine signaling that results in tumor growth and survival. ANGPT2: Angiopoietin 2; IGF: insulin-like growth factor; IGF-1R: IGF-1 receptor; VEGFR2*P: VEGFR2 receptor with rs1870377 A/T or A/A polymorphisms.

In conclusion, CD38 upregulation after IC blockade therapy may contribute to the development of hyperprogression through the release of high levels of adenosine into the TME and the consequent activation of the ADORA2a pathway, which may lead to tumor insensitivity to IFN-γ action, to the downregulation of p53 with consequent tumor growth, and to strong immunosuppression. CD38 upregulation may also be an adaptive immune response to a hyperactivated immune setting induced by ICI therapy. In this context, CD38 may promote the apoptosis of effector T cells via the AICD process, leading to a protumorigenic setting. Finally, CD38 may enhance hypoxia signaling pathways in tumor cells or endothelial cells, leading to increased angiogenesis, immunosuppression and tumor proliferation (Figure 3).

## 4. Conclusions

HPD is surely a challenge in the context of atypical therapeutic responses to ICI therapy. In this review, we focused on two factors that often recur in the landscape of clinical resistance to ICI: the cytokine IFN-γ and the alternative immune checkpoint CD38. We have discussed the detrimental role that IFN-γ secretion and CD38 upregulation can play in the TME after ICIs, and have suggested that a possible interplay exists between these factors, in the context of resistance to therapy and HPD. 

Based on the reported data, patients with a high risk of developing hyperprogression may have specific traits, such as mutations on the PD-L1 intracytoplasmic domain and molecules involved in downstream inflammasome signaling pathways. Increased levels of secreted IFN-γ during ICI therapy may further stimulate this activated path, thus inducing the formation of an inflammatory microenvironment that stimulates CD38 upregulation. The combined activation of the inflammasome pathway, which leads to substantial MDSC recruitment, and CD38 activation, which reduces the sensitivity of tumor cells to IFN-γ through ADORA2a activation, may lead to a loss of therapeutic efficacy and uncontrolled tumor progression. Moreover, in some patients, ICI therapy may, at first, hyperactivate the antitumor immune response, either because of tumor-intrinsic characteristics or differential responses of T cells to treatment. Immune hyperactivation may trigger negative feedback mechanisms that have the aim of preventing excessive immune responses, and thus lead to the upregulation of CD38 and the activation of the AICD mechanism, which can be induced both by IFN-γ and by CD38 itself. Hyperprogressive patients, who have been reported to experience AICD, have displayed a dampening of effector T-cell responses and consequent tumor progression. 

Finally, an important role is played by the immunosuppressive responses induced by IFN-γ and CD38 through the induction of IDO1 and hypoxic pathways, respectively. Mutations that are associated with the constitutive expression of IDO1, the activation of oncogenic pathways and HIF-1α expression have been reported in hyperprogressive patients and may play a role in HPD. An increase in angiogenesis and the recruitment of immunosuppressive cells, such as M2-like macrophages and Treg cells, serve as a framework for this hypothesized HPD mechanism.

As only a few targeted studies on the role of IFN-γ and CD38 in HPD mechanisms have been conducted, new effort should be invested in collecting further data about these two factors and their interplay. The chance to treat HPD patients with CD38 inhibitors is a real opportunity to overcome tumor acceleration. The correlation between CD38 and angiogenesis also suggests the possible use of anti-angiogenic inhibitors in HPD patients. IFN-γ signature/key mediators could also be investigated and monitored over time in order to prevent AICD. The immune infiltrate of re-biopsies should also be monitored and compared to the baseline to quantify the presence of immunosuppressive populations. Moreover, the evaluation of the genetic signature of each tumor and the identification of polymorphisms associated with immune response in patients, especially those involved in IFN-γ and CD38-dependent mechanisms, may provide a relevant tool to identify patients at risk of developing HPD.

## Figures and Tables

**Table 1:** The incidence of hyperprogression after ICI therapy and definitions of HPD in different studies.

Authors	Tumor Types	Treatment	Rate of HPD (%)	Criteria Used to Define HPD
Champiat et al. (2017) [12]	Multiple tumor types ^1^	PD-1/PD-L1 inhibitors	9.2	RECIST-defined progressive disease at first evaluation TGR ratio ≥ 2 (on-treatment versus before treatment)
Kanjanapan et al. (2019) [18]	Multiple tumor types ^2^	CTLA-4, PD-1/PD-L1 inhibitors	7	
Aoki et al. (2019) [9]	AGC	PD-1 inhibitors	29.4	>2 fold increase in TGR (compared with prior therapy)
Ferrara et al. (2018) [13]	NSCLC	PD-1/PD-L1 inhibitors	14	RECIST-defined progressive disease at first evaluation ΔTGR increase > 50% at first evaluation
Saâda-Bouzid et al. (2017) [14]	HNSCC	PD-1/PD-L1 inhibitors	29.4	TGK ratio ≥ 2 (on-treatment versus before treatment)
Kim et al. (2019) [15]	NSCLC	PD-1/PD-L1 inhibitors	17	
Sasaki et al. (2019) [10]	AGC	PD-1 inhibitors	21	TGK ratio ≥ 2 (on-treatment versus before treatment) >50% increase in tumor burden (compared to pre-immunotherapy)
Kato et al. (2017) [19]	Multiple tumor types ^3^	CTLA-4, PD-1/PD-L1 inhibitors	3.9	TTF < 2 months >50% increase in tumor burden (compared to pre-immunotherapy) >2 fold increase in progression pace
Kamada et al. (2019) [11]	AGC	PD-1 inhibitors	10	
Matos et al. (2018) [20]	Multiple tumor types ^4^	PD-1/PD-L1 inhibitors	15	TTF < 2 months increase in lesions ≥ 10 mm plus: (A) ≥40% increase in tumor burden (compared to pre-immunotherapy) or (B) ≥20% increase in tumor burden plus multiple new lesions
Lo Russo et al. (2019) [21]	NSCLC	PD-1/PD-L1 inhibitors	25.7	TTF < 2 months increase of 50% in the sum of target lesions major diameters between baseline (B) and first radiologic evaluation (R) appearance of ≥2 new lesions in an organ already involved (between B and R) spread of the disease to a new organ (between B and R) decrease in ECOG-PS ≥ 2 during the first 2 months of treatment

^1^ Melanoma, renal carcinoma, colorectal cancer, urothelial cancer, hepatocellular carcinoma, head and neck carcinoma, cutaneous squamous cell carcinoma, breast cancer, ovarian cancer, glioblastoma endometrium cancer, glioblastoma, cervix cancer, gastric and esophagus cancer, mesothelioma, pancreas cancer, sarcoma. ^2^ Head and neck carcinoma, gynecological tumors, lung cancer, gastrointestinal tumors, genitourinary tumors, melanoma, sarcoma. ^3^ Melanoma, non-small-cell lung cancer, squamous cell carcinoma of head and neck, cutaneous squamous cell carcinoma, renal cell carcinoma, and colorectal cancer. ^4^ Melanoma, lung cancer, breast cancer, colon cancer, others. HPD—Hyperprogressive disease; NSCLC—Non-small-cell lung cancer; HNSCC—Head and neck squamous cell carcinoma; AGC—Advanced Gastric Cancer; RECIST — Response Evaluation Criteria in Solid Tumors; TGR—Tumor growth rate; ΔTGR — TGR on treatment minus the TGR before treatment; TGK—tumor growth kinetics; TTF—time-to-treatment failure; ECOG-PS—Eastern Cooperative Oncology Group performance status.

## Data Availability

No new data were created or analyzed in this study. Data sharing is not applicable to this article.
